# Some Consequences of the War

**Published:** 1915-09-04

**Authors:** 


### SOME CONSEQUENCES OF THE WAR.

The war is an event so penetrating and commanding that to detach one's thoughts from it, and to realise that the usual activities of national life have in many respects to be continued, are tasks not easily accomplished. This is difficult enough in reference to the arrangements and duties of the moment, and to undertakings which have a prospect near at hand. It is still more difficult in regard to plans which mean years of preparation and which look for maturity to a relatively remote future. Who is bold enough nowadays to assume with confidence the prophet's mantle or to say in what respects the social and economic life of Europe, or even of our own country, will be modified by the consequences of the great struggle of the nations? That there will be changes is certain, and these changes will touch many issues. But to define the outlook in set terms and with precision is beyond the reach of human endeavour. Not less true is it that the compelling force of circumstances continues to demand the erection of plans and the choice of decisions based upon an expectation, uncertain though it be, of the course of future events. On the large scale such plans and decisions engage the statesman, the politician, and the student of economic law, who are compelled to-day to advise policies of which the consequences will only appear years ahead. A similar task in reference to his own fate is not infrequently prescribed for the individual citizen, and such responsibility is particularly conspicuous in the case of those younger members of the community to whom time has brought in this involved moment the choice of a career. In any circumstances, so important a step engages sympathy and consideration for those who needs must take it. But to-day these qualities are specially summoned, seeing that the choice has to be made under unusually unstable conditions and amidst influences which render judgment difficult to command. Youth, in spite of the war, has to face the great decision and to select its goal; and those of us who have realised in experience the inevitable consequences of such election would fain offer a word of forbearing and helpful counsel.

One general remark may be made relative to the special difficulties of the hour. It is that the modifying and healing influences of time tend ever to put back order even in places where chaos for the moment seems predominant. War is not a normal but an abnormal condition of the nations. That peace will return is certain, and not less certain is it that, though much will be wrecked and changed by the struggle, long-established habits of thought and action will re-assert themselves and the movement of the world will resume its ordered course. It is necessary to avoid exaggeration in this respect, and to remember that ere many years have passed the horrors and calamities and confusions of the present situation will be replaced by peaceful activities, and that the lives even of the belligerent nations will be resumed on a more reasonable plane. The hope and anticipation which in this country we confidently cultivate is that, however terrible the loss, one outcome of the struggle will be relief from the sense of impending peril which has so long brooded over Europe. Hence, however severe the economic handicap, we count on the probability of more freedom of the spirit and a larger mental life alike for the individual and for the nation. These general considerations apply with special force to those who are at this moment debating the possible selection of medicine as a profession.

